# Balsacone C, a New Antibiotic Targeting Bacterial Cell Membranes, Inhibits Clinical Isolates of Methicillin-Resistant *Staphylococcus aureus* (MRSA) Without Inducing Resistance

**DOI:** 10.3389/fmicb.2019.02341

**Published:** 2019-10-15

**Authors:** Héloïse Côté, André Pichette, François Simard, Marie-Eve Ouellette, Lionel Ripoll, Mouadh Mihoub, Doria Grimard, Jean Legault

**Affiliations:** ^1^Laboratoire d’Analyse et de Séparation des Essences Végétales, Département des Sciences Fondamentales, Université du Québec à Chicoutimi, Chicoutimi, QC, Canada; ^2^Centre de Recherche sur la Boréalie, Université du Québec à Chicoutimi, Chicoutimi, QC, Canada; ^3^Laboratoire de Microbiologie, Complexe Hospitalier de la Sagamie, Chicoutimi, QC, Canada

**Keywords:** *Populus balsamifera*, buds, balsacone, antibiotic, *Staphylococcus aureus*, MRSA

## Abstract

New options are urgently needed for the treatment of methicillin-resistant *Staphylococcus aureus* (MRSA) infections. Balsacone C is a new dihydrochalcone extracted from *Populus balsamifera* that has been reported previously as being active against *Staphylococcus aureus*. Here, we evaluate the antibacterial activity of balsacone C against MRSA. Thirty-four (34) MRSA isolates were obtained from hospitalized patients; these isolates were then characterized for their resistance. Most of these MRSA (>85%) were resistant to penicillin, amoxicillin/clavulanic acid, ciprofloxacin, moxifloxacin, levofloxacin, clindamycin, erythromycin, and cefoxitin as well as being sensitive to linezolid, trimethoprim/sulfamethoxazole, rifampicin, and gentamicin. When tested against all MRSA isolates and various gram-positive bacteria, the antibacterial activity of balsacone C produced a MIC of 3–11.6 mg/mL. We observed no resistant isolates of MRSA (against balsacone C) even after 30 passages. Microscopy fluorescence showed that bacteria cell membrane integrity was compromised by low concentrations of balsacone C. Scanning electron microscope (SEM) confirmed balsacone C–provoked changes in the bacterial cell membrane and we find a dose-dependent release of DNA and proteins. This loss of cellular integrity leads to cell death and suggests a low potential for the development of spontaneous resistance.

## Introduction

Infectious diseases are now the second leading global cause of death in the world. Across the globe, bacterial infections kill 700,000 people annually (World Health Organization [WHO], 2016), and by 2050, deaths attributable to antibiotic-resistant infections may even exceed cancer-related deaths (Bagnoli et al., 2017). Methicillin-resistant *Staphylococcus aureus* (MRSA) infections remain a primary cause of infection-related mortality and represent a major global health care problem. This antibiotic-resistant bacteria was first identified within a health care setting around 1960, and it emerged in the community in the early 1990s (Vestergaard et al., 2019). Methicillin resistance in *Staphylococcus aureus* is due to the acquisition of the mobile genetic element SCCmec (mecA or mecC). This gene codes for a penicillin-binding protein (PBP) that makes the strain resistant to all beta-lactam antibiotics (Bagnoli et al., 2017). Since *S. aureus* has a notorious ability to acquire or develop resistance to antibiotics, it is considered to be a global pandemic threat (CDC, 2013; Sergelidis and Angelidis, 2017). The rapidly increasing limitations of vancomycin and teicoplanin as primary therapies for severe and life-threatening infections have also raised concerns (Gardete and Tomasz, 2014). Within the past 20 years, MRSA has developed reduced susceptibility to vancomycin-intermediate *Staphylococcus aureus* (VISA), and complete resistance has emerged (VRSA) (Bal et al., 2017). Moreover, MRSA resistance to linezolid and daptomycin has also been documented (McGuinness et al., 2017). Novel antibiotics are required urgently to combat this life-threatening pathogen (Laxminarayan et al., 2014; Assis et al., 2017; Subramani et al., 2017).

Over the last 30 years, the gap has widened between the emergence of antibiotic-resistant strains and the development of new antibiotics. Because of this disparity, there is heightened interest in finding new bioactive compounds derived from plants (Subramani et al., 2017). The widespread use of antibiotics derived from fungal or bacterial origin since the 1950s has limited interest in the study of plant compounds as potential antimicrobial agents. Nevertheless, most plants in natural settings respond to fungal and bacterial pathogens by activating low molecular weight antimicrobial compounds (Taylor, 2013). Subramani et al. (2017) list 15 plant-derived compounds and 40 plant extracts that demonstrate antibacterial activities against various multidrug-resistant pathogens, including MRSA.

Several plant species in the Canadian boreal forest show promise as sources of new antibiotics. A new class of potential antibiotics has been recovered recently from buds of *Populus balsamifera* L. (Lavoie et al., 2013). *P. balsamifera* (Salicaceae) is a hardwood tree having a widespread distribution in eastern North America. Buds from this species were used frequently by First Nation populations as treatment for a range of health problems, from the common cold to diabetes (Moerman, 1998). The use of *P. balsamifera* buds within different types of preparations that were applied to wounds, cuts, frostbite, and insect bites suggests that these buds may possess antibacterial properties.

In a previous study, we showed that *P. balsamifera* buds extract had antibacterial activity against a single strain of methicillin-sensitive *Staphylococcus aureus* (MSSA). We also isolated for the first time the Balsacones A, B and C and elucidated their structures. The Balsacone C (BC) (C_24_H_22_O_5_) is a new dihydrochalcone derivative from *P. balsamifera* buds and was found to be one of the most potent compounds (Lavoie et al., 2013). In the present study, we characterize a suite of clinical MRSA isolates obtained from patients hospitalized at the Chicoutimi Hospital, Saguenay, QC, Canada. We then evaluate the antibacterial activity of BC (Supplementary Figure S1) against MRSA and various gram-positive and gram-negative bacterial strains. In addition, we assess the propensity of MRSA to develop resistance to BC. Finally, in this work, we investigate the action mechanism responsible for the antibacterial activity of BC and assess its cytotoxicity on human normal cells.

## Materials and Methods

### Cell Culture

We obtained human skin fibroblasts WS1 (ATCC CRL-1502) from the American Type Culture Collection (Manassas, VA, United States). Cells were grown in a humidified atmosphere at 37°C in 5% CO_2_, in Dulbecco’s Minimum Essential Medium supplemented with 10% fetal calf serum (Hyclone, Logan, UT, United States), 1 × solution of sodium pyruvate, 1 × vitamins, 1 × non-essential amino acids, 100 IU of penicillin and 100 μg/ml streptomycin (Cellgro^®^, Mediatech, Manassas, VA, United States).

### Isolation and Characterization of Methicillin-Resistant *S. aureus* Isolates

Thirty-four (34) isolates of MRSA and one (1) MSSA were obtained from nares (*n* = 31), throat (*n* = 3) and groin pus (*n* = 1) from patients hospitalized at the Chicoutimi Hospital. We identified *S. aureus* using a Slidex Staph-Kit (bioMerieux Vitek, Inc., Hazelwood, MO, United States). Quality control was performed with MRSA isolates ATCC 43300 and *S. aureus* strain ATCC 25923. We determined the *S. aureus* profile using an API Staph test strip having a saline bacterial suspension having a 0.5 McFarland standard (bioMerieux, Durham, NC, United States). API Staph strips were left for 24 h before the results were read. For all isolates, we ran a latex agglutination (Slidex) test to detect methicillin resistance in Staphylococci – based on the production of low-affinity PBP2a, which is encoded by the mecA or mecC gene (data not shown).

### Antibiotic Susceptibility Using the Disk Diffusion Method

We produced an antibiogram to confirm the identification of MRSA isolates using a disk diffusion test (*Kirby-Bauer*) as described by the National Committee for Clinical Laboratory Standards (NCCLS) (Clinical Laboratory Standard Institute [CLSI], 2019). The tested antibiotics included: penicillin (10 units), amoxicillin/clavulanic acid (20/10 μg), ciprofloxacin (5 μg), moxifloxacin (5 μg), levofloxacin (5 μg), clindamycin (15 μg), erythromycin (15 μg), cefoxitin (30 μg), linezolid (30 μg), trimethoprim/sulfamethoxazole (1.25/23.75 μg), rifampicin (5 μg), gentamicin (10 μg), and vancomycin (30 μg). The cultures were first inoculated on a non-selective plate for 18–24 h. We collected each colony and then transferred the individual colonies to a 5-ml tryptic-soy broth. The inoculum density was standardized with BaSO_4_ using 0.5 McFarland standard, and all isolates were incubated at 35°C. We then inoculated the Muller-Hinton agar plates. After 15 min, we applied the disks having a fixed concentration of antibiotics to the plate surface. Plates were incubated at 35°C for 16–24 h. We measured the growth inhibition zone around each disk, and we related the diameter of the zone to the bacteria susceptibility. The results of the disk diffusion test are expressed as susceptible, intermediate, or resistant. We used *S. aureus* ATCC 25923 as our quality control organism.

### Compound and Bacterial Strains

Lavoie et al. (2013) describe the isolation of BC. Purity was confirmed by ^1^H-NMR (proton magnetic resonance spectroscopy used to determine the structure of a molecule). Antimicrobial activities of BC were tested against *Escherichia coli* (ATCC 25922), *S. aureus* (ATCC 25923), *Enterobacter aerogenes* (C3032834), *Enterobacter cloacae* (B9040334), *Salmonella typhimurium* (C6162763), *Burkholderia cepacia* (C6101997), *Klebsiella pneumonia* (B8302928), *Staphylococcus epidermidis* (B9030482), *Enterococcus faecalis* (ERV), and *Listeria monocytogenes* (B8222880). All bacterial strains were provided by the Chicoutimi Hospital, Saguenay, QC, Canada. *Streptococcus uberis* (CL) was provided by Collège Laflèche, Trois-Rivières, QC, Canada.

### Antibacterial Activity Measurements Using the Microdilution Method

We tested the antibacterial activities of BC using the microdilution method of Banfi et al. (2003). Briefly, 50 μL of growing bacteria were plated in 96-well plates (Costar, Corning Inc.) in nutrient broth (Difco). Increasing concentrations of BC (diluted in methanol) were then added. The final concentration of methanol in the culture medium was maintained at 0.1% (v/v) to avoid solvent toxicity. The negative control was a bacterial suspension without treatment, and the blank consisted of a culture medium only. We tested the bacterial suspension plus solvent to demonstrate the absence of solvent toxicity. Concentration of 3.5 × 10^5^ CFU/mL was used. Microplates were incubated for 24 h at 37°C, and the absorbance was then measured at 540 nm using an automated Varioskan Ascent plate reader. Results are expressed as the concentration at which 100% of bacterial growth is inhibited (MIC).

### Induction of Resistance Against BC and Rifampicin

To induce the emergence of drug resistance, we used the broth-dilution procedure of Dalhoff et al. (2005). Briefly, approximately 3.5 × 10^5^ CFU was added to 10 ml nutrient broth. Bacteria were grown overnight in nutrient broth containing different concentrations of the compounds (1–10 mg/L for BC and 0.004– mg/L for rifampicin). For each passage (passage refers to transferring bacteria from a previous culture to a fresh growth medium), we transferred an aliquot (1:20) from the tube containing the highest drug concentration with visible bacterial growth to a second set of serial antibiotic dilutions. After incubating the broths overnight, we repeated the dilution procedure for a total period of 10 passages for rifampicin and 30 passages for BC. After completion of the passages, we subcultured the bacteria having the highest MIC onto a drug-free nutrient broth. We tested these latter samples for antibiotic susceptibility with microdilution assay. Results are expressed as the concentration inhibiting 50% of bacterial growth (IC_50_).

### Cell Membrane Integrity

To evaluate the integrity of cell membranes, we used the BacLight Live/Dead bacterial viability kit (L-7012; Molecular Probes). Briefly, we added approximately 2 × 10^7^ CFU/ml to tubes containing 10 ml of nutrient broth. Untreated and treated samples having BC (IC_50_: 1.5 mg/L and MIC: 3 mg/L) were grown for 24 h at 37°C and then were centrifuged at 10,000 × *g* for 10 min. The supernatant was removed, and the pellet was resuspended in 2 ml NaCl (0.85%). We added 1 ml of the sample to 20 ml NaCl (0.85%), incubated the suspension for 1 h at room temperature, and then centrifuged the mixture at 10,000 × *g* for 10 min (repeated twice). We removed the supernatant and resuspended the pellet in 10 ml NaCl. We added 3 μL of Syto9 and 3 μL of propidium iodide (PI) to each sample. Samples were then incubated at room temperature for 15 min. We analyzed the integrity of the bacterial membranes using a fluorescence microscope (Reichert) equipped with a halogen lamp, Neoplan 100 × /1.25 oil objective and a 1,713 filter cube (fluorescein; 490/510/520 nm) at 1,000× magnification. In the assays, the fluorescent green nucleic acid stain Syto9 passes in living and dead bacterial cells, whereas PI cannot penetrate intact membranes. When the cellular membrane is damaged, PI can penetrate bacteria and cause the cells to appear red (31).

### Scanning Electron Microscope

Previous work has shown that membrane damage can be confirmed by combining fluorescence microscopy and scanning electron microscope (SEM) imagery (De Sousa et al., 2012; Nostro et al., 2017). We prepared the samples for SEM following De Sousa et al. (2012) with some modifications. Briefly, untreated and treated samples having BC (1.5 and 3 mg/L) were grown 3 h at 37°C. We removed the samples from the cultures, washed them with PBS and then fixed the samples in phosphate buffers (pH 7.2) containing 2.5% glutaraldehyde for 2 h at room temperature. The fixed cells were collected via centrifugation at 2,000 × *g* and washed three times with phosphate buffers. The fixed bacteria were dehydrated with ethanol (30–95%). We mounted the dried specimens on aluminum stubs using a conductive carbon cement; the specimens were allowed to dry and were then coated with a gold film. We observed the samples with an SEM at 20 kV and 25,000× magnification.

### Release of Bacterial Intracellular Constituents

Intracellular material released from the cells was quantified as described in Virto et al. (2005) with some modifications. Briefly, we added approximately 8 × 10^7^ CFU/ml to tubes containing 10 ml nutrient broth. Untreated and treated samples having BC (1.5 and 3 mg/L) were grown 3 h at 37°C and were then centrifuged at 2,000 × *g* for 10 min. We transferred the supernatant in a cuvette and measured the UV absorbance using a spectrophotometer (Multiskan^TM^ GO Spectrophotometer – Thermo Fisher Scientific) – nucleic acids have an absorption peak at 260 nm, proteins at 280 nm. We compared our results with those of untreated control samples.

### Acridine Orange/Ethidium Bromide Staining

We evaluated the integrity of WS1 cell membranes using a double staining assay with acridine orange (AO) and ethidium bromide (EB) as described in Ribble et al. (2005). AO (15 mg) and EB (50 mg) were dissolved in 1 ml of 95% ethanol and then added to 49 ml of PBS, gently mixed, aliquoted, and stocked at –20°C. Before use, we diluted the stock solution 1/10 in PBS (pH 7.4). We plated growing WS1 cells (1 × 10^4^ cells) onto a 96-well plate and incubated them for 18 h. After treating the cells with 1.5 and 3 mg/L of BC, we re-incubated them for 14 h. The cells were then washed with PBS and incubated for 5 min with the dual fluorescent staining solution (AO/EB). For our observations, we used Cytation3 (Cell Imaging Multi-Mode Reader) at excitation and emission wavelengths of 530 and 590 nm, respectively.

### Calcein-AM Cell Cytotoxicity Assay

We tested the toxicity of BC against the human healthy cell line WS1 as described in Yang et al. (2002). In brief, we plated WS1 cells (1 × 10^4^ cells) onto a 96-well plate and incubated the plates for 18 h. After the incubation period, we treated the cells with 1.5 and 3 mg/L of BC and 0.82 mg/L of doxorubicin. After 24 h of incubation, we removed the media and washed the cells with PBS. We then added 100 μL of calcein-AM (0.25 mg/L) and incubated the cells for 20 min. WS1 cells were washed twice in PBS, and we observed the cells using Cytation3 (Cell Imaging Multi-Mode Reader) at excitation and emission wavelengths of 490 and 520 nm, respectively. First, we evaluated the effect of BC on WS1 membranes using double labeling with OA and EB (Mihoub et al., 2018). Human healthy cells were incubated in the presence or absence of BC at IC_50_ (1.5 mg/L) and MIC (3 mg/L) concentrations. Beta-hederin, a cytolytic triterpenoid, was used as positive control.

### Statistical Analysis

For all analyses, we ran two-way ANOVAs all followed by a post-test Holm-Sidak method using SigmaStat^®^ software (Systat Software Inc., San Jose, CA, United States). Differences were deemed as statistically significant when *P* < 0.05.

## Results and Discussion

All 35 bacterial clinical isolates were indeed *S. aureus*. Moreover, API Staph identified two different *S. aureus* biotypes including the biotype 6736153 and the biotype 6736113 at a probability of 97.8 and 86.7%, respectively (Supplementary Table S1). Our control *S. aureus* ATCC 25923 was identified as the biotype 6736153. A latex agglutination test for the PBP2a protein encoding by mecA genes also confirmed that thirty-four (34) bacterial isolated were MRSA (data not shown). An antibiogram of *S. aureus* and all isolates was performed with various classes of antibiotics – they include beta-lactam (penicillin, amoxicillin/clavulanic acid), fluoroquinolone (ciprofloxacin, moxifloxacin, levofloxacin), lincosamide (clindamycin), macrolide (erythromycin), cephalosporin (cefoxitin), oxazolidone (linezolid), sulfonamide (trimethoprim/sulfamethoxazole), rifamycin (rifampicin), aminoglycoside (gentamicin) and glycopeptide (vancomycin). The results indicated that *S. aureus* (ATCC 25923) was sensitive to all antibiotics tested except levofloxacin (Supplementary Tables S2, S3). Moreover, with the exception of isolate 08-U-0189, all MRSA were resistant to penicillin, erythromycin, and cefoxitin. Consequently, isolate 08-U-0189 is considered as a MSSA. On the other hand, all isolates were 91.4% resistant to ciprofloxacin, moxifloxacin, levofloxacin, and clindamycin and 88.5% resistant to amoxicillin/clavulanic acid (Supplementary Table S2). These antibiogram profiles were similar to the typical phenotype of endemic MRSA isolates as experienced by Chao et al. (2013) in China. Interestingly, three MRSA were sensitive to all fluoroquinolone antibiotics (08-U-0194, 08-U-0204, and 08-U-0209). Moreover, 3% of MRSA were resistant to TMP/SMX and rifampicin, 6% to gentamicin and 34% had intermediate resistance to vancomycin, however, the MIC should be determined to confirm the presence of a VISA (Supplementary Table S3). All MRSA were sensitive to linezolid. The most resistant isolate of MRSA (08-U-0214), showed resistance to beta-lactam (penicillin, amoxicillin/clavulanic acid, methicillin), fluoroquinolone (ciprofloxacin, moxifloxacin, levofloxacin), lincosamide (clindamycin), macrolide (erythromycin), cephalosporin (cefoxitin), rifamycin (rifampicin), aminoglycoside (gentamicin), and intermediate to glycopeptide (vancomycin).

The antibacterial activity of BC, was evaluated using a microdilution assay against *S. aureus* (ATCC 25923) and the 35 MRSA and MSSA isolates. BC was active against all bacteria tested producing a MIC of 3–11.7 mg/L (Table 1). Furthermore, the most resistant MRSA (08-U-0214) was also sensitive to BC and had a MIC of 3.9 mg/L. Most of the new antibiotic candidates reported in the literature possess a MIC in the same range of BC. For example, Adnani et al. (2017) describe keyicin, a new a bis-nitroglycosylated anthracycline, that has an action mechanism different from that of other anthracyclines. Keyicin was active against gram-positive *Bacillus subtilis* with a MIC of 7.97 mg/L and against MRSA with a MIC of 2.01 mg/L. Zhang et al. (2017) obtained MICs of 1.39–5.57 mg/L for two compounds from the Aurachin family that had antimicrobial activity against *S. aureus*, *Streptococcus pyogenes*, and *B. subtilis*. They obtained a MIC of 44.57 mg/L against MRSA, whereas rifampicin and ampicillin had respective MICs of 3.29 and 22.36 mg/L.

**TABLE 1:** Evaluation of antibacterial activity of balsacone C against *Staphylococcus aureus* and the clinical isolates of MRSA and MSSA.

**Isolate number**	**Balsacone C**	**Isolate number**	**Balsacone C**
	**MIC (mg/L)**		**MIC (mg/L)**
ATCC 25923	3.4 ± 0.7	08-U-0202	6.4 ± 0.8
08-U-0185	12 ± 1	08-U-0203	5.1 ± 0.6
08-U-0186	4.1 ± 0.6	08-U-0204	4.7 ± 0.6
08-U-0187	8 ± 1	08-U-0205	3.4 ± 0.5
08-U-0188	4 ± 1	08-U-0206	4 ± 1
08-U-0189	6.7 ± 0.7	08-U-0207	3 ± 1
08-U-0190	6.9 ± 0.4	08-U-0208	7.8 ± 0.4
08-U-0191	6 ± 1	08-U-0209	4.9 ± 0.7
08-U-0192	11 ± 1	08-U-0213	11.6 ± 0.5
08-U-0193	7.4 ± 0.5	08-U-0214	3.9 ± 0.1
08-U-0194	5.6 ± 0.6	08-U-0215	4.4 ± 0.3
08-U-0195	8.6 ± 0.7	08-U-0216	9.4 ± 0.7
08-U-0196	4.2 ± 0.6	08-U-0217	4.6 ± 0.3
08-U-0197	3.4 ± 0.2	08-U-0218	5.4 ± 0.1
08-U-0198	3 ± 1	08-U-0219	3.6 ± 0.4
08-U-0199	7.6 ± 1.0	08-U-0220	3.3 ± 0.6
08-U-0200	10 ± 1	08-U-0221	4.9 ± 0.6
08-U-0201	3.3 ± 0.6	08-U-0222	3.5 ± 0.5

*ATCC 25923 is *Staphylococcus aureus*. Data are representative of three different experiments. Main ± standard deviation, *n* = 3. MIC is defined as the lowest concentration able to inhibit 100% of bacterial growth.*

**FIGURE 1:**
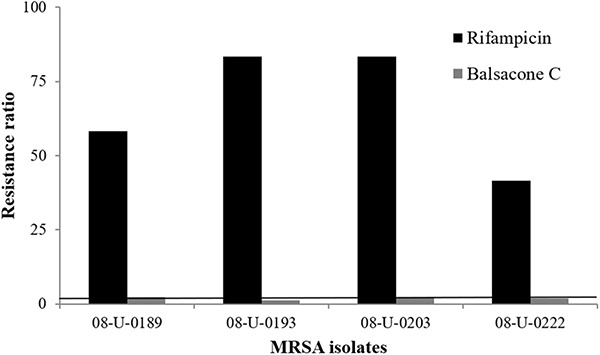
Induced bacterial resistance using rifampicin and balsacone C on four different MRSA isolates. Resistance ratio is IC_50_ of resistant isolate/IC_50_ of susceptible isolate (>30 fold) after 10 passages for rifampicin and 30 passages for balsacone C. The solid line defines the limits of equal susceptibility (onefold).

We tested BC activity against four gram-positive (*S. epidermidis*, *S. uberis*, *E. faecalis*, and *L. monocytogenes*) and six gram-negative bacteria (*B. cepacia*, *E. aerogenes*, *E. cloacae*, *E. coli*, *K. pneumonia*, and *S. typhimurium*). BC inhibited the bacterial growth of all gram-positive bacteria with a MIC of 10.1–32 mg/L (Table 2). In contrast, BC was inactive against the gram-negative taxa with a MIC > 100 mg/L. The exception was for *B. cepacia* with a MIC of 32 mg/L. *B. cepacia* is naturally resistant to some antibiotic classes including polymyxins, aminoglycosides, trimethoprim, chloramphenicol, quinolones, and beta-lactams (Sousa et al., 2011). The intrinsic multidrug resistance of this bacteria occurs due to the presence of various enzymes and efflux pumps that remove antibiotics from the cell (De Soyza et al., 2008). Rushton et al. (2013) suggested membrane charge/ionization influences antibiotic binding and resistance. Interestingly, these resistance mechanisms of *B. cepacia* are not efficient against BC suggesting an alternative action mechanism is at work. Firstly, we investigated the BC action mechanism using a model of resistance induction described by Dalhoff et al. (2005). Using rifampicin as a positive control, the resistance of one MSSA (08-U-0189) and three MRSA (08-U-0193; 08-U-0203; 08-U-0222) isolates was achieved easily (Figure 1). Indeed, the IC_50_ of rifampicin for all tested isolates was initially lower than 0.012 mg/L, while after ten passages of MRSA in the presence of sub-MIC levels of rifampicin, the isolates were at least 30 × more resistant with an IC_50_ of 0.41 ± 0.07 to 1.00 ± 0.02 mg/L. Using the same approach, we attempted to induce BC resistance. The IC_50_ of BC was first 1.4 ± 0.1 to 2.9 ± 0.5 mg/L, however, repeated passages (30) of MRSA in the presence of sub-MIC levels of BC failed to produce resistant bacteria with an IC_50_ of 3.0 ± 0.3 to 3.9 ± 0.4 mg/L after induction.

**TABLE 2:** Evaluation of antibacterial activity of balsacone C against different bacteria.

**Bacteria taxa**			**Antibacterial activity of balsacone C**
	**Strain**	**Gram**	**MIC (mg/L)**
*Enterococcus faecalis*	ERV	+	10.9 ± 0.8
*Listeria monocytogenes*	B8222880	+	20 ± 3
*Staphylococcus epidermidis*	B9030482	+	27 ± 2
*Streptococcus uberis*	CL	+	10.1 ± 0.7
*Burkholderia cepacia*	C6101997	−	32 ± 2
*Enterobacter aerogenes*	C3032834	−	>100
*Enterobacter cloacae*	B9040334	−	>100
*Escherichia coli*	ATCC 25922	−	>100
*Klebsiella pneumonia*	B8302928	−	>100
*Salmonella typhimurium*	C6162763	−	>100

*Data are representative of three different experiments. Main ± standard deviation, *n* = 3. MIC is defined as the lowest concentration able to inhibit 100% of bacterial growth.*

**FIGURE 2:**
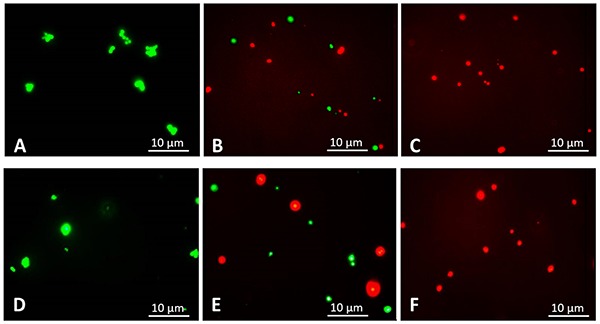
Fluorescence microscopic images, 1,000× magnification. Following 3 h of treatment with balsacone C, *Staphylococcus aureus* and MRSA (08-U-0214) were stained using a LIVE/DEAD BacLight Viability kit. Live cells are indicated by green fluorescence (Syto 9), whereas cells having damaged membranes are indicated by red fluorescence (PI). Untreated *S. aureus* (negative control) **(A)**; *S. aureus* after treatment with 1.5 mg/L balsacone C **(B)**; *S. aureus* after treatment with 3 mg/L BC **(C)**; untreated MRSA (negative control) **(D)**; MRSA after treatment with 1.5 mg/L balsacone C **(E)**; and MRSA after treatment with 3 mg/L balsacone C **(F)**.

The rapid bactericidal action of BC suggests that the possible action mechanism might occur via altering the integrity of bacterial cell membranes, as suggested by Ling et al. (2015) with there study on a new antibiotic, the teixobactin. The effect of BC on the membrane integrity, assessed using the dead/live BacLight bacterial viability assay (Grégori et al., 2001; Stiefel et al., 2015), found untreated *S. aureus* (control) to have no membrane damage (Figure 2A). *S. aureus* treated with 1.5 mg/L BC (IC_50_) generated a mix of green fluorescent (intact membrane) and red fluorescent (damaged membrane) bacteria (Figure 2B), and all bacteria had damaged membranes at 3 mg/L (MIC) (Figure 2C). We obtained similar results with MRSA (Figures 2D–F). Therefore, BC alters bacterial cell membranes.

**FIGURE 3:**
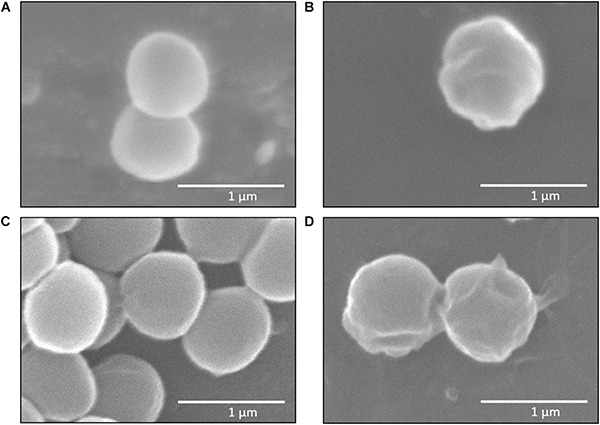
Scanning electron microscope (SEM) images of *Staphylococcus aureus* and MRSA (08-U-0214), 25,000× magnification. Untreated *S. aureus* (negative control) **(A)**; *S. aureus* after treatment with 3 mg/L balsacone C **(B)**; untreated MRSA (negative control) **(C)**; and MRSA after treatment with 3 mg/L balsacone C **(D)**.

Structural changes in the membrane, such as an altered fluidity, should lead to a slight modification in the cell surface structure (Alakomi et al., 2006). SEM images of samples having a 3-h exposure to BC at a MIC concentration (3 mg/L) determined if BC affected bacterial cell membrane structures and possibly membrane functions (Sipponen et al., 2009). We observed marked alterations of the cell structure surface of *S. aureus* (Figure 3B) and MRSA (Figure 3D) compared to the negative control (Figures 3A,C). Cell surfaces became irregular, and we observed invagination and structural alterations. These SEM observations of *S. aureus* and MRSA cell surfaces confirm the susceptibility of these bacteria to BC.

**FIGURE 4:**
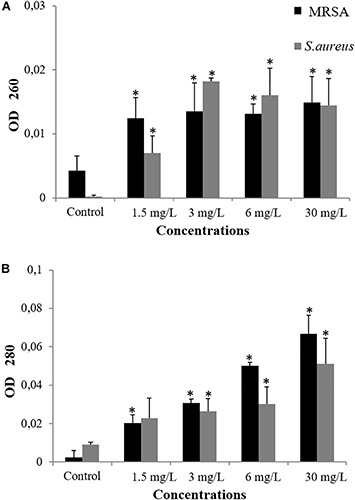
Measurement of cellular leakage of nucleic acid **(A)** and protein **(B)** from *S. aureus* and MRSA (08-U-0214) after 3 h of exposure to balsacone C. Experiments were performed at a cell density of 8 × 10^7^ CFU/ml following exposure to 1.5, 3, 6, and 30 mg/L balsacone C. Control represents the untreated cells. For all data *n* = 3. ^∗^Significantly different from control (*P* < 0.05).

Damage induced by antibacterial agents such as BC can provoke the release of intracellular components – these components include small ions, such as potassium and phosphates, and much larger molecules, including DNA, RNA, and proteins (Maillard, 2002; Johnston et al., 2003; Liu et al., 2004; Virto et al., 2005; Lee and Je, 2013; Ukuku et al., 2013; He et al., 2016; McKenzie et al., 2016; Sannasiddappa et al., 2017). To test whether BC provoked the release of DNA and proteins, we observed the UV-absorbance values at 260 and 280 nm. For treated samples of *S. aureus* and MRSA, absorbance values increased significantly in a dose-dependent manner when compared to untreated cells (*P* < 0.006). Thus, BC appears to induce the disruption of the cellular membrane and cause the release of intracellular constituents such as DNA (Figure 4A) and proteins (Figure 4B). Furthermore, the BC concentrations that induce cell membrane damages match the observed antibacterial activity.

Some antibiotics are known to target bacterial cell membranes. Epand et al. (2016) reviewed compounds that interact with bacterial cell membranes. They mention that this approach for antibiotics is a complex field that is only beginning to be exploited. Antibiotics that target the bacterial membrane or precursors appear to have a high potential as they show a fast and extensive bactericidal effect, in particular against MRSA and VISA (Bambeke et al., 2008). Membrane-damaging antibiotics can interact directly with the bacterial membrane bilayer, thereby disrupting its function and its physical integrity, which leads to the loss of membrane permeability and the altering of membrane properties. For example, daptomycin induces membrane permeabilization, depolarization, and disruption of multiple cellular processes; telavancin and oritavancin inhibit peptidoglycan biosynthesis by binding to the D-Ala-D-Ala termini to cause membrane permeabilization and depolarization, whereas polymyxin and chlorhexidine cause a collapse of the membrane potential (Kuyyakanond and Quesnel, 1992; Hurdle et al., 2011). Although we observed that BC appears to target bacterial cell membranes, further research is needed to determine the specific target of BC within the bacterial cell.

**FIGURE 5:**
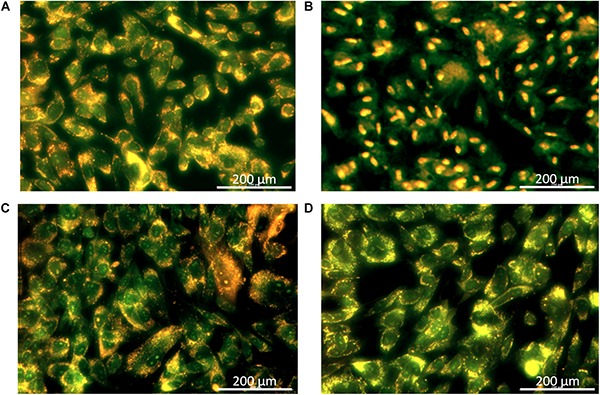
Cellular membrane integrity after treatments with balsacone C; images were captured by a Cytation 3 Cell Imaging Multi-Mode Reader with a 20× objective. WS1 cells were stained with acridine orange/ethidium bromide to visualize intact membranes. Stained control cells **(A)**; positive control beta-hederine 7.5 mg/L **(B)**; cell treated with 1.5 mg/L balsacone C **(C)**; and cell treated with 3 mg/L balsacone C **(D)**. Data are representative of three different experiments.

**FIGURE 6:**
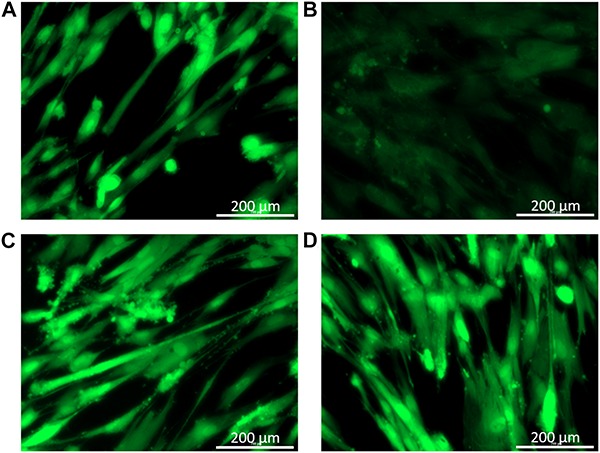
Cell viability after treatments with balsacone C; images were captured by a Cytation 3 Cell Imaging Multi-Mode Reader with a 20× objective. WS1 cells were stained with calcein-am to visualize living (green) and dead cells. Stained control cells **(A)**; positive control doxorubicine 0.55 mg/L **(B)**; cell treated with 1.5 mg/L balsacone C **(C)**; and cell treated with 3 mg/L balsacone C **(D)**. Data are representative of three different experiments.

Balsacone C was found previously weakly cytotoxic against human cells using viability assay (Lavoie et al., 2013). In the present work, the effect of BC on human cellular membrane was assessed on healthy skin fibroblasts, WS1. Beta-hederin, a membrane cell permeabilizer, was used as positive control (Gauthier et al., 2009; Mihoub et al., 2018). In contrast to untreated cells (Figure 5A), beta-hederin treated cells are permeable to EB. This produces a red-orange fluorescence located at the nucleus (Figure 5B), which indicates membrane alteration (Mihoub et al., 2018). The healthy human cells treated with 1.5 and 3 mg/L (MIC) of BC produced a green-orange fluorescence diffused throughout the cell without nucleus fluorescence. A similar pattern was observed in the untreated cells (Figures 5C,D) and confirms that BC does not cause any membrane alteration at both concentrations tested. The viability of WS1 healthy cells, post-treatment with BC, was evaluated using calcein-AM. This hydrophobic probe permeates live cells easily and becomes strongly green fluorescent after cleavage of the acetoxymethyl ester by intracellular esterases. As expected, untreated cells had an intense green fluorescence (Figure 6A). Cells treated with BC at 1.5 and 3 mg/L (Figures 6C,D) also produced an intense green fluorescence, thereby confirming that BC is not cytotoxic for WS1 human cells. In contrast, the green fluorescence of cells treated with 0.82 mg/L of doxorubicin (Figure 6B) was much weaker. These preliminary results suggest that BC could be used to treat bacterial infections without affecting healthy human cells.

## Conclusion

New antibiotics are urgently warranted to combat resistant bacteria such as MRSA. Our results indicate that BC induces bacterial cell membrane damage. This damage leads to the loss of membrane integrity and the release of intracellular constituents, followed by cell death after relatively short incubation times. This promising new therapeutic candidate represents a “membrane active agent” mainly used against gram-positive bacteria, such as MRSA. Induction experiments on MRSA and MSSA isolates did not lead to resistance. Future research will focus on improving the structure of balsacone in order to increase its activity and identify the specific target of BC. Moreover, *in vivo* tests on mice models should be performed to determine the best way of administration, the toxicity and the efficacy of BC.

## Data Availability Statement

The raw data supporting the conclusion of this manuscript will be made available by the authors, without undue reservation, to any qualified researcher.

## Author Contributions

HC performed the experiments, analyzed the data, and wrote the manuscript. FS, M-EO, MM, and LR performed some of the experiments and revised the manuscript. AP and DG analyzed the data and revised the manuscript. JL conceived the experiments, analyzed the data, and wrote the manuscript.

## Conflict of Interest

The authors declare that the research was conducted in the absence of any commercial or financial relationships that could be construed as a potential conflict of interest.
