# Supplementary material for: Balsacone C, a New Antibiotic Targeting Bacterial Cell Membranes, Inhibits Clinical Isolates of Methicillin-Resistant Staphylococcus aureus (MRSA) Without Inducing Resistance
Source: Front Microbiol. 2019 Oct 15;10:2341. doi: 10.3389/fmicb.2019.02341 (PMC6804428; doi:10.3389/fmicb.2019.02341)
Supplement: Supplementary file 1 [file Data_Sheet_1.PDF]

Balsacone C, a new antibiotic targeting bacterial cell membranes, inhibits clinical isolates of methicillin-resistant *Staphylococcus aureus* (MRSA) without inducing resistance

Héloise Coté[1], André Pichette[1], François Simard[1], Marie-Eve Ouellette[1], Lionel Ripoll[1], Mouadh Mihoub[1], Doria Grimard[2] and Jean Legault[1*]

[1]Laboratoire d'analyse et de séparation des essences végétales, Département des sciences fondamentales. Université du Québec à Chicoutimi, Chicoutimi, Québec, Canada.

[2]Laboratoire de Microbiologie, Complexe Hospitalier de la Sagamie, Chicoutimi, Québec, Canada.

[*] Correspondence:

Jean Legault

Jean.Legault@uqac.ca

KEYWORDS: *Populus balsamifera*, buds, balsacone, antibiotic, *Staphylococcus aureus*, MRSA

Supplementary Table 1. Identification and sources of clinical isolates of *Staphylococcus aureus*.

Isolate number	Date of sampling	Source/origin	Bacterial identity[b]	Strain profile[c]	Probability of identity[c] (%)
08-U-0185	2007-03-05	Nares	S. aureus	6736153	97.8
08-U-0186	2007-03-05	Nares	S. aureus	6736153	97.8
08-U-0187	2007-03-05	Nares	S. aureus	6736153	97.8
08-U-0188	2007-03-05	Nares	S. aureus	6736153	97.8
08-U-0189	2007-03-12	Throat	S. aureus	6736153	97.8
08-U-0190	2007-03-12	Nares	S. aureus	6736153	97.8
08-U-0191	2007-03-12	Nares	S. aureus	6736153	97.8
08-U-0192	2007-03-12	Nares	S. aureus	6736153	97.8
08-U-0193	2007-03-14	Nares	S. aureus	6736153	97.8
08-U-0194	2007-03-19	Nares	S. aureus	6736153	97.8
08-U-0195	2007-03-19	Nares	S. aureus	6736113	86.7
08-U-0196	2007-03-19	Nares	S. aureus	6736113	86.7
08-U-0197	2007-03-26	Nares	S. aureus	6736113	86.7
08-U-0198	2007-03-28	Nares	S. aureus	6736113	86.7
08-U-0199	2007-04-10	Nares	S. aureus	6736113	86.7
08-U-0200	2007-04-11	Throat	S. aureus	6736113	86.7
08-U-0201	2007-04-11	Nares	S. aureus	6736113	86.7
08-U-0202	2007-04-16	Groin pus	S. aureus	6736113	86.7
08-U-0203	2007-04-30	Nares	S. aureus	6736153	97.8
08-U-0204	2007-05-09	Nares	S. aureus	6736153	97.8
08-U-0205	2007-05-09	Nares	S. aureus	6736113	86.7
08-U-0206	2007-05-09	Nares	S. aureus	6736113	86.7
08-U-0207	2007-05-09	Nares	S. aureus	6736113	86.7
08-U-0208	2007-05-09	Nares	S. aureus	6736113	86.7
08-U-0209	2007-05-09	Nares	S. aureus	6736113	86.7
08-U-0213	2008-05-09	Nares	S. aureus	6736153	97.8
08-U-0214	2008-05-09	Nares	S. aureus	6736153	97.8
08-U-0215	2008-05-09	Nares	S. aureus	6736113	86.7
08-U-0216	2008-05-09	Nares	S. aureus	6736113	86.7
08-U-0217	2008-05-09	Throat	S. aureus	6736153	97.8
08-U-0218	2008-05-09	Nares	S. aureus	6736113	86.7
08-U-0219	2008-05-09	Nares	S. aureus	6736153	97.8
08-U-0220	2008-05-09	Nares	S. aureus	6736113	86.7
08-U-0221	2008-05-09	Nares	S. aureus	6736153	97.8
08-U-0222	2008-05-09	Nares	S. aureus	6736113	86.7
ATCC 25923	(-)[a]	ATCC	S. aureus	6736153	97.8

[a]Unknown

[b]Slidex Staph-Kit

[c]API Staph test strip

Supplementary Table 2. Antibiogram of clinical isolates of MRSA using the disk diffusion test with various classes of antibiotics including beta-lactam (penicillin-PEN, amoxicillin/clavulanic acid-AMC), fluoroquinolone (ciprofloxacin-CIP, moxifloxacin-MXF, levofloxacin-LVX), lincosamide (clindamycin-CLI), macrolide (erythromycin-ERY) and cephalosporin (cefoxitin-FOX)

Isolate number	PEN[a]	AMC[b]	CIP[c]	MXF[d]	LVX[e]	CLI[f]	ERY[g]	FOX[h]
8-U-0185	Ø (R)	15 (R)	Ø (R)	10 (R)	Ø (R)	Ø (R)	Ø (R)	11 (R)
08-U-0186	Ø (R)	14 (R)	Ø (R)	Ø (R)	Ø (R)	Ø (R)	Ø (R)	11 (R)
08-U-0187	Ø (R)	15 (R)	Ø (R)	10 (R)	Ø (R)	Ø (R)	Ø (R)	10 (R)
08-U-0188	Ø (R)	12 (R)	Ø (R)	Ø (R)	Ø (R)	Ø (R)	Ø (R)	10 (R)
08-U-0189	Ø (R)	29 (S)	Ø (R)	Ø (R)	Ø (R)	Ø (R)	Ø (R)	30 (S)
08-U-0190	Ø (R)	16 (R)	Ø (R)	10 (R)	Ø (R)	18 (S)	Ø (R)	12 (R)
08-U-0191	Ø (R)	15 (R)	Ø (R)	10 (R)	Ø (R)	Ø (R)	Ø (R)	11 (R)
08-U-0192	Ø (R)	13(R)	Ø (R)	10 (R)	Ø (R)	Ø (R)	Ø (R)	Ø (R)
08-U-0193	Ø (R)	15 (R)	Ø (R)	10 (R)	Ø (R)	Ø (R)	Ø (R)	11 (R)
08-U-0194	13 (R)	16 (R)	25 (S)	29 (S)	28 (S)	Ø (R)	Ø (R)	14 (R)
08-U-0195	Ø (R)	18 (R)	Ø (R)	11 (R)	Ø (R)	Ø (R)	Ø (R)	11 (R)
08-U-0196	9 (R)	15 (R)	Ø (R)	10 (R)	Ø (R)	16 (I)	Ø (R)	11 (R)
08-U-0197	Ø (R)	14 (R)	Ø (R)	11 (R)	Ø (R)	Ø (R)	Ø (R)	Ø (R)
08-U-0198	10 (R)	18 (R)	Ø (R)	10 (R)	Ø (R)	Ø (R)	Ø (R)	11 (R)
08-U-0199	Ø (R)	16 (R)	Ø (R)	10 (R)	Ø (R)	Ø (R)	Ø (R)	12 (R)
08-U-0200	Ø (R)	15 (R)	Ø (R)	Ø (R)	Ø (R)	Ø (R)	Ø (R)	11 (R)
08-U-0201	Ø (R)	14 (R)	Ø (R)	10 (R)	Ø (R)	Ø (R)	Ø (R)	11 (R)
08-U-0202	Ø (R)	14 (R)	Ø (R)	10 (R)	Ø (R)	Ø (R)	Ø (R)	10 (R)
08-U-0203	21 (R)	22 (S)	Ø (R)	Ø (R)	Ø (R)	17 (S)	Ø (R)	15 (R)
08-U-0204	10 (R)	15 (R)	26 (S)	30 (S)	26 (S)	Ø (R)	Ø (R)	11 (R)
08-U-0205	Ø (R)	15 (R)	Ø (R)	10 (R)	Ø (R)	Ø (R)	Ø (R)	11 (R)
08-U-0206	Ø (R)	14 (R)	Ø (R)	Ø (R)	Ø (R)	Ø (R)	Ø (R)	11 (R)
08-U-0207	Ø (R)	14 (R)	Ø (R)	Ø (R)	Ø (R)	Ø (R)	Ø (R)	Ø (R)
08-U-0208	Ø (R)	14 (R)	Ø (R)	10 (R)	Ø (R)	Ø (R)	Ø (R)	Ø (R)
08-U-0209	14 (R)	19 (S)	26 (S)	30 (S)	29 (S)	Ø (R)	Ø (R)	14 (R)
08-U-0213	Ø (R)	14 (R)	Ø (R)	10 (R)	Ø (R)	Ø (R)	Ø (R)	Ø (R)
08-U-0214	10 (R)	15 (R)	Ø (R)	10 (R)	Ø (R)	Ø (R)	Ø (R)	13 (R)
08-U-0215	Ø (R)	13 (R)	Ø (R)	Ø (R)	Ø (R)	Ø (R)	Ø (R)	Ø (R)
08-U-0216	Ø (R)	11 (R)	Ø (R)	Ø (R)	Ø (R)	Ø (R)	Ø (R)	Ø (R)
08-U-0217	Ø (R)	14 (R)	Ø (R)	Ø (R)	Ø (R)	Ø (R)	Ø (R)	Ø (R)
08-U-0218	10 (R)	16 (R)	Ø (R)	10 (R)	Ø (R)	Ø (R)	Ø (R)	13 (R)
08-U-0219	Ø (R)	17 (R)	Ø (R)	10 (R)	Ø (R)	Ø (R)	Ø (R)	Ø (R)
08-U-0220	Ø (R)	14 (R)	Ø (R)	10 (R)	Ø (R)	Ø (R)	Ø (R)	Ø (R)
08-U-0221	10 (R)	14 (R)	Ø (R)	10 (R)	Ø (R)	Ø (R)	Ø (R)	Ø (R)
08-U-0222	15 (R)	22 (S)	Ø (R)	10 (R)	Ø (R)	Ø (R)	Ø (R)	14 (R)
ATCC 25923	33 (S)	33 (S)	28 (S)	31 (S)	Ø (R)	26 (S)	28 (S)	27 (S)
MRSA resistant (%)	100	88.5	91.4	91.4	91.4	91.4	100	97.1

[a-h]The results present the diameter of inhibition growth around each disk (mm).

[a]Penicillin (10 units): resistant (R) ≤28; susceptible (S) ≥29.

[b]Amoxicillin/clavulanic acid (20/10 µg): resistant (R) ≤19; susceptible (S) ≥20.

[c]Ciprofloxacin (5 µg): resistant (R) ≤15; intermediate (I) 16–20; susceptible (S) ≥21.

[d]Moxifloxacin (5 µg): resistant (R) ≤20; intermediate (I) 21–23; susceptible (S) ≥24.

[e]Levofloxacin (5 µg): resistant (R) ≤15; intermediate (I) 16–18; susceptible (S) ≥19.

[f]Clindamycin (2 µg): resistant (R) ≤14; intermediate (I) 15–16; susceptible (S) ≥17.

[g]Erythromycin (15 µg): resistant (R) ≤13; intermediate (I) 14–22; susceptible (S) ≥23.

[h]Cefoxitin (30 µg): resistant (R) ≤21; susceptible (S) ≥22.

Supplementary Table 3. Antibiogram of clinical isolates of MRSA using disk diffusion tests with various classes of antibiotics including oxazolidone (linezolid-LZD), sulfonamide (trimethoprim/sulfomethoxazole-SXT), rifamycin (rifampicin-RIF), aminoglycoside (gentamicin-GEN) and glycopeptide (vancomycin-VAN)

Isolate number	LZD[a]	SXT[b]	RIF[c]	GEN[d]	VAN[e]
08-U-0185	30 (S)	34 (S)	33 (S)	22 (S)	20 (S)
08-U-0186	26 (S)	32 (S)	30 (S)	19 (S)	19 (S)
08-U-0187	31 (S)	32 (S)	30 (S)	21 (S)	19 (S)
08-U-0188	28 (S)	30 (S)	28 (S)	20 (S)	17 (I)
08-U-0189	28 (S)	33 (S)	31 (S)	20 (S)	20 (S)
08-U-0190	26 (S)	32 (S)	31 (S)	21 (S)	19 (S)
08-U-0191	27 (S)	35 (S)	33 (S)	21 (S)	19 (S)
08-U-0192	28 (S)	32 (S)	31 (S)	20 (S)	17 (I)
08-U-0193	28 (S)	32 (S)	32 (S)	21 (S)	19 (S)
08-U-0194	27 (S)	31 (S)	31 (S)	10 (R)	19 (S)
08-U-0195	28 (S)	33 (S)	28 (S)	21 (S)	19 (S)
08-U-0196	28 (S)	33 (S)	32 (S)	20 (S)	18 (I)
08-U-0197	28 (S)	33 (S)	32 (S)	20 (S)	17 (I)
08-U-0198	28 (S)	33 (S)	32 (S)	21 (S)	18 (I)
08-U-0199	27 (S)	33 (S)	29 (S)	20 (S)	19 (S)
08-U-0200	29 (S)	33 (S)	31 (S)	21 (S)	19 (S)
08-U-0201	28 (S)	32 (S)	28 (S)	19 (S)	18 (I)
08-U-0202	28 (S)	33 (S)	32 (S)	20 (S)	19 (S)
08-U-0203	27 (S)	33 (S)	31 (S)	21 (S)	18 (I)
08-U-0204	27 (S)	30 (S)	28 (S)	10 (R)	17 (I)
08-U-0205	30 (S)	34 (S)	31 (S)	20 (S)	19 (S)
08-U-0206	27 (S)	33 (S)	31 (S)	20 (S)	19 (S)
08-U-0207	27 (S)	33 (S)	32 (S)	20 (S)	19 (S)
08-U-0208	27 (S)	33 (S)	30 (S)	20 (S)	19 (S)
08-U-0209	27 (S)	31 (S)	28 (S)	21 (S)	19 (S)
08-U-0213	27 (S)	33 (S)	31 (S)	21 (S)	18 (I)
08-U-0214	27 (S)	31 (S)	Ø (R)	Ø (R)	18 (I)
08-U-0215	27 (S)	Ø (R)	31 (S)	20 (S)	20 (S)
08-U-0216	27 (S)	31 (S)	28 (S)	25 (S)	19 (S)
08-U-0217	26 (S)	33 (S)	30 (S)	21 (S)	19 (S)
08-U-0218	26 (S)	33 (S)	23 (S)	20 (S)	18 (I)
08-U-0219	27 (S)	31 (S)	23 (S)	20 (S)	18 (I)
08-U-0220	28 (S)	33 (S)	31 (S)	18 (S)	19 (S)
08-U-0221	27 (S)	31 (S)	28 (S)	25 (S)	20 (S)
08-U-0222	28 (S)	32 (S)	30 (S)	24 (S)	19 (S)
ATCC 25923	28 (S)	32 (S)	32 (S)	23 (S)	19 (S)
MRSA resistant (%)	0	2.9	2.9	8.6	0

[a-e]The results present the diameter of inhibition growth around each disk (mm).

[a]Linezolid (30 µg): susceptible (S) ≥21.

[b]Trimethoprim/sulfomethoxazole (1.25/23.75 µg): resistant (R) ≤10; intermediate (I) 11–15; susceptible (S) ≥16.

[c]Rifampicin (5 µg): resistant (R) ≤16; intermediate (I) 17–19; susceptible (S) ≥20.

[d]Gentamicin (10 µg): resistant (R) ≤12; intermediate (I) 13–14; susceptible (S) ≥15.

[e]Vancomycin (30 µg): resistant (R) ≤15; intermediate (I) 16–18; susceptible (S) ≥19.

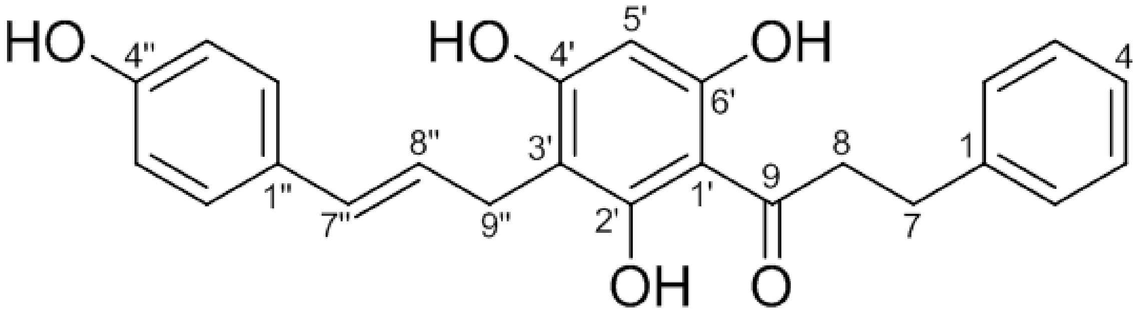

Balsacone C

Supplementary Figure 1.
